# Umbilical cord-derived mesenchymal stromal cell therapy to prevent the development of neurodevelopmental disorders related to low birth weight

**DOI:** 10.1038/s41598-023-30817-3

**Published:** 2023-03-07

**Authors:** Masahiro Tsuji, Takeo Mukai, Yoshiaki Sato, Yasue Azuma, Saki Yamamoto, Florence Cayetanot, Laurence Bodineau, Atsuto Onoda, Tokiko Nagamura-Inoue, Jacques-Olivier Coq

**Affiliations:** 1 Department of Food and Nutrition, Kyoto Women’s University, 35 Kitahiyoshi-cho, Imakumano, Higashiyama-ku, Kyoto, 605-8501 Japan; 2 Department of Cell Processing and Transfusion, Institute of Medical Science, The University of Tokyo, Tokyo, Japan; 3 Division of Neonatology, Center for Maternal-Neonatal Care, Nagoya University Hospital, Nagoya, Japan; 4 Institut National de la Santé et de la Recherche Médicale (Inserm), UMR_S1158 Neurophysiologie Respiratoire Expérimentale et Clinique, Sorbonne Université, Paris, France; 5 Centre National de la Recherche Scientifique (CNRS), Institut des Sciences du Mouvement (ISM) UMR7287, Aix Marseille Université, 163 avenue de Luminy, CC 910, 13288 Marseille Cedex 09, France

**Keywords:** Stem cells, Diseases, Neurology

## Abstract

Low birth weight (LBW) increases the risk of neurodevelopmental disorders (NDDs) such as attention-deficit/hyperactive disorder and autism spectrum disorder, as well as cerebral palsy, for which no prophylactic measure exists. Neuroinflammation in fetuses and neonates plays a major pathogenic role in NDDs. Meanwhile, umbilical cord-derived mesenchymal stromal cells (UC-MSCs) exhibit immunomodulatory properties. Therefore, we hypothesized that systemic administration of UC-MSCs in the early postnatal period may attenuate neuroinflammation and thereby prevent the emergence of NDDs. The LBW pups born to dams subjected to mild intrauterine hypoperfusion exhibited a significantly lesser decrease in the monosynaptic response with increased frequency of stimulation to the spinal cord preparation from postnatal day 4 (P4) to P6, suggesting hyperexcitability, which was improved by intravenous administration of human UC-MSCs (1 × 10^5^ cells) on P1. Three-chamber sociability tests at adolescence revealed that only LBW males exhibited disturbed sociability, which tended to be ameliorated by UC-MSC treatment. Other parameters, including those determined via open-field tests, were not significantly improved by UC-MSC treatment. Serum or cerebrospinal fluid levels of pro-inflammatory cytokines were not elevated in the LBW pups, and UC-MSC treatment did not decrease these levels. In conclusion, although UC-MSC treatment prevents hyperexcitability in LBW pups, beneficial effects for NDDs are marginal.

## Introduction

The incidence of neurodevelopmental impairments in children with fetal growth restriction (FGR), which is also known as intrauterine growth restriction (IUGR), with resultant low birth weight (LBW, < 2500 g) is approximately 25%, while the incidence of neurodevelopmental impairments in children without FGR is only approximately 5%^1^. Worldwide, LBW occurs in approximately 14.6% of infants; these infants exhibit a higher risk of developing neurodevelopmental disorders (NDDs), such as attention-deficit/hyperactivity disorder and autism spectrum disorder (ASD)^2,3^. LBW children may exhibit combinations of neurological disorders with varying severities, as LBW is associated with a higher prevalence of cerebral palsy (5–8%), motor and cognitive delays, and mental health disorders^4,5^.

Etiological factors related to perinatal brain injury in preterm or LBW infants include exposure to systemic hypoxia, ischemia, infection/inflammation, hyperoxia, excessive stimuli, pain, stress, and/or neurotoxic drugs^6–8^. In previous studies, we developed an FGR and LBW rat model by exposure to mild intrauterine hypoperfusion (MIUH) on embryonic day 17 (E17), which led to diffuse brain damage characterized by hypomyelination, axonal degeneration, astrogliosis, enlarged lateral ventricles, and neuronal loss in various brain regions including the hippocampal complex^9–12^. We found significantly increased levels of inflammation-related chemokines and ischemia-related proteins in the placentas. This tendency was also observed in the brains of fetuses, although it was not significant^10,12^. In recent studies, signs of inflammation were confirmed in rat models with comparable exposures to MIUH^13–15^. Moreover, adult rats with MIUH-induced LBW exhibited hyperactivity, impaired sensory input integration, memory deficits, minor postural and locomotor deficits, anatomical and functional disorganization, and hyperexcitability in the sensorimotor circuitry^10–12,16,17^. This hyperexcitability is possibly related to the expression of K^+^–Cl^−^ cotransporter type 2 (KCC2), the main chloride cotransporter in the central nervous system (CNS) that regulates chloride homeostasis and cell excitability, and consequently, behavior and memory^18–20^. Thus, MIUH seemingly induces perinatal inflammation, which could cause the main symptoms observed in LBW infants who develop NDDs. Recent postmortem studies have revealed a major impact of neuroinflammation on the development of brain damage and neurological disorders in LBW infants^6,21–23^.

To our knowledge, there are no effective therapies for brain damage associated with LBW. Therapeutic hypothermia (33–35 °C) is now the standard modality of care for near-term and term neonates with clear signs of hypoxic-ischemic encephalopathy but not for neonates born before 36 weeks of gestation^8^. Other therapies, including the administration of magnesium sulfate or erythropoietin, are targeted in preclinical trials but appear to have no significant protective effect in preterm or LBW infants^24^. Mesenchymal stromal cells (MSCs) produce cytokines and neurotrophic factors that suppress inflammation and enhance neurogenesis and angiogenesis^25–27^. The umbilical cord (UC) is an excellent source of MSCs, and the collection method is non-invasive. Furthermore, UC-derived MSCs (UC-MSCs) possess robust immunomodulatory and paracrine properties^28,29^. We recently reported that LBW rats postnatally treated with UC-MSCs exhibited better performance in negative geotaxis and rotarod tests and less neuronal loss in the cerebral cortex compared to non-treated LBW rats^27^. In the present study, we administered UC-MSCs on postnatal day 1 (P1) to LBW pups exposed to MIUH at E17 and assessed the presence of abnormalities, focusing on behaviors associated with NDDs (open-field activity and social interactions), post-activation depression (indicative of normal excitability in the spinal network), body growth, inflammation in the plasma and cerebrospinal fluid (CSF), and neuronal counts in the hippocampus.

## Results

We used 85 LBW rats subjected to MIUH and 56 sham-operated rats of either sex; all rats were operated on at E17. Thirty-nine LBW rats and 26 sham pups received intravenous injection of MSCs on P1, and 46 LBW and 30 sham rats were intravenously injected with a vehicle solution on P1. The rats from all four experimental groups (sham-vehicle, sham-MSC, LBW-vehicle and LBW-MSC) underwent the same assessments of body weight, behavioral performance including negative geotaxis (P6 to P8), brain weight, and neuronal counts (7 weeks of age). As behaviors may differ between males and females at puberty and later life stages, behavioral tests performed from 3 to 7 weeks of age (i.e., the open-field test and three-chamber sociability test, as well as body weight and brain weight tests) were separately analyzed in males and females. Males and females in the sham-MSC groups were excluded from these behavioral test analyses due to the low number of males in this group (*n* = 3). We evaluated cytokine expression (P2), electrophysiological post-activation depression (P5 to P6), and KCC2 expression in the lumbar spinal cord (P8) using Western blotting in different sets of experimental rats for the four groups.

### Uterine blood flow

**Figure 1:**
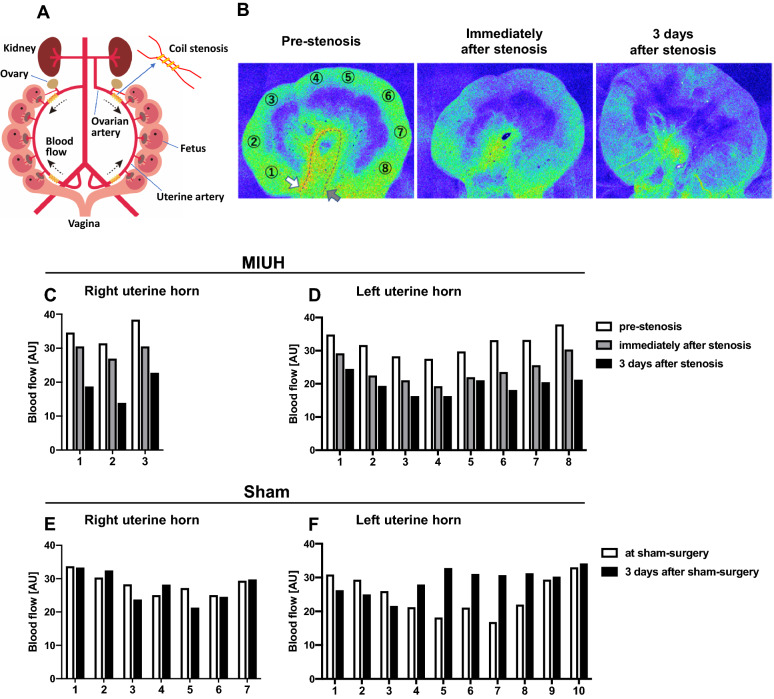
Uterine blood flow. Schema of the arteries supplying blood to the uterus and the coil installation process (**A**). Representative images of blood flow in the left uterine horn, as measured using laser speckle flowmetry, in a dam with microcoil stenosis. The white arrow indicates the site of coil stenosis in the ovarian artery, while the gray arrow indicates the site of the coil stenosis in the uterine artery (**B**). Blood flow levels in a dam with microcoil stenosis (**C**,**D**) and in a dam with sham surgery (**E**,**F**). AU, arbitrary unit; MIUH, mild intrauterine hypoperfusion.

We measured uterine blood flow via laser speckle flowmetry during the surgery, by wrapping microcoils on bilateral ovarian and uterine arteries (Fig. 1A–D), and the sham surgery (Fig. 1E,F) on E17. A reexamination was done 3 days post-surgery, i.e., on E20 (Fig. 1B–F). In dams with microcoils, uterine blood flow decreased immediately after artery stenosis, which persisted on reexamination. In dams with sham surgery, no such reduction in blood flow was observed on reexamination. The part of the uterine artery close to either the tip of the uterine horn or the vagina presented with relatively higher blood flow, whereas the in-between part of the uterine artery had relatively lower blood flow.

### Body weight and brain weight

**Figure 2:**
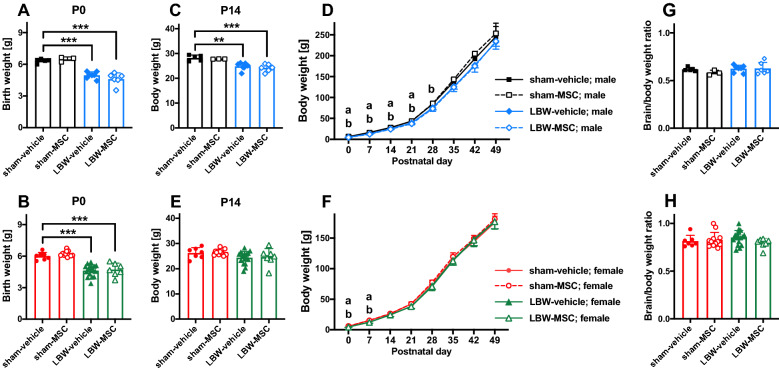
Body weight. Birth weights of male (**A**) and female (**B**) pups. Body weight at P14 of male (**C**) and female (**E**) pups. Overall temporal changes, from P0 to P49, in males (**D**) and females (**F**). Ratio of brain weight to body weight at the time of perfusion-fixation, i.e., at P56, in males (**G**) and females (**H**). In the male group: sham-vehicle (*n* = 5), sham-MSC (*n* = 3), LBW-vehicle (*n* = 7), and LBW-MSC (*n* = 6). In the female group: sham-vehicle (*n* = 7), sham-MSC (*n* = 12), LBW-vehicle (*n* = 17), and LBW-MSC (*n* = 7). **p < 0.01, ***p < 0.001. a: *p* < 0.05, mice in the sham-vehicle vs. LBW-vehicle groups. b: *p* < 0.05, mice in the sham-vehicle vs. LBW-MSC groups. Mean ± standard deviation. LBW, low birth weight; MSC, mesenchymal stromal cell; P0, postnatal day 0 (the day of birth); P14, postnatal day 14.

The mean birthweight of pups born to sham-operated dams was 6.33 ± 0.20 g in males and 6.00 ± 0.36 g in females (Fig. 2A,B). The majority of pups born to dams with MIUH exhibited LBW, which was defined as a birth weight < 5.5 g; pups weighing 5.5 g or more were excluded from this study. The overall temporal changes from P0 to P49 differed significantly among the males in the four groups (sham-vehicle, sham-MSC, LBW-vehicle, and LBW-MSC) (F_3, 17_ = 5.57, *p* = 0.0076) (Fig. 2C,D). Males in the LBW-vehicle and LBW-MSC groups had a significantly lower body weight than those in the sham-vehicle group from P0 up to P28. P35 onward, weight did not differ between males in the LBW and sham-vehicle groups. The overall temporal changes from P0 to P49 did not differ among the females in the four groups (F_3, 39_ = 1.578, *p* = 0.210) (Fig. 2E,F). The body weight of females in the LBW-vehicle and LBW-MSC groups matched those of females in the sham-vehicle group by P14 (Fig. 2E,F). The body weights did not significantly differ between male and female pups in the sham-vehicle and sham-MSC groups at any age.

Ratios of brain weight to body weight at the time of perfusion-fixation (P56) did not differ among males (F_3, 17_ = 0.837, *p* = 0.492) and females (F_3, 39_ = 1.26, *p* = 0.300) in the four groups (Fig. 2G,H).

### Early inhibition in the lumbar spinal cord

**Figure 3:**
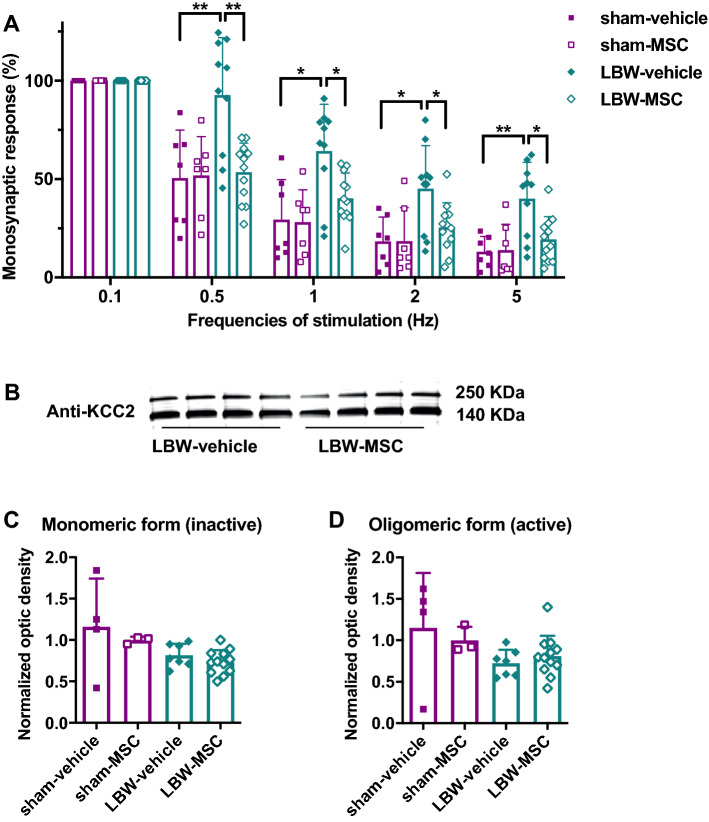
Early excitability in the lumbar spinal cord. (**A**) The relative monosynaptic responses of the motor neurons recorded in the ventral root decreased with increasing frequencies of stimulation of the dorsal root in the four groups of pups from P5 to P6, compared to the reference level at stimulation at a frequency of 0.1 Hz (*p* < 0.0001). Lower reductions in the relative monosynaptic responses were noted for the LBW-vehicle group, compared to the sham-vehicle group, at all frequencies of stimulation after the reference one (0.1 Hz), which is indicative of lumbar network hyperexcitability. These responses in pups of the LBW-MSC group were significantly reduced, compared to pups in the LBW-vehicle group at all frequencies, which suggests a reduction of hyperexcitability in the lumbar spinal cord with MSC treatment. Sham-vehicle (*n* = 7), sham-MSC (*n* = 7), LBW-vehicle (*n* = 10), and LBW-MSC (*n* = 12). (**B**) Representative images of Western blot analysis of the monomeric and oligomeric forms from the total fraction of KCC2 in LBW pups treated using a vehicle or MSCs at P8. (**C**) Quantified expression levels of the monomeric (inactive) form of KCC2. There seemed to be decreases in LBW pups compared to pups in sham pups, but the differences were not significant. (**D**) Quantified expression levels of the oligomeric (active) form of KCC2. There were no significant differences among the groups. Sham-vehicle (*n* = 4), sham-MSC (*n* = 3), LBW-vehicle (*n* = 7), and LBW-MSC (*n* = 12). *p < 0.05, **p < 0.01. Mean ± standard deviation. KCC2, K^+^–Cl^−^ cotransporter type 2; LBW, low birth weight; MSC, mesenchymal stromal cell; P5, postnatal day 5.

To determine the early impact of MIUH on the functional reorganization and excitation/inhibition balance in rat pups, we assessed the alterations in the monosynaptic reflex loop using an in vitro whole spinal cord preparation from P4 to P6^16^. This procedure is comparable to the stretch or Hoffmann reflex assessment in early stages. Repeated measures one-way analysis of variance (ANOVA) revealed significant effects in both groups (F_3, 32_ = 27.05, *p* < 0.0001) and increased stimulation frequencies (F_4, 32_ = 4136.81, *p* < 0.0001), as well as a significant interaction between groups and stimulation frequencies (F_12, 32_ = 2.23, *p* = 0.013), indicative of differential reductions in monosynaptic responses at increased frequencies depending on the group. For example, pups in the LBW-vehicle group exhibited a different response profile compared to other groups (Fig. 3A). In pups of the sham-vehicle group, the amplitude of the monosynaptic response was gradually depressed when the dorsal root was repeatedly stimulated using frequencies that ranged from 0.1 to 5 Hz (Fig. 3A), which is comparable to the in vitro post-activation depression observed in pups of the non-treated sham group and representative of standard excitability in the lumbar spinal cord^16^. In contrast, the gradual depression in monosynaptic response with increasing stimulation frequencies was significantly decreased in pups of the LBW-vehicle group compared to pups of the sham-vehicle group at every examined frequency (Fig. 3A), suggesting hyperexcitability within the lumbar spinal cord and early signs of spasticity, as previously found^16^. In pups of the sham-MSC and LBW-MSC groups, the depression in monosynaptic responses with increasing stimulation frequencies was comparable to that found in pups of the sham-vehicle group. As hypothesized, the monosynaptic responses in pups of the LBW-MSC group were significantly reduced compared to pups of the LBW-vehicle group at increasing frequencies of 0.5, 1, 2, and 5 Hz (post-hoc comparisons: 0.015 < *p* < 0.0006) (Fig. 3A), which is indicative of reduced hyperexcitability after early MSC treatment in pups of the LBW group.

### Early expression of KCC2

KCC2, which contributes to regulate chloride homeostasis and cell excitability, is a K-Cl cotransporter in the main chloride extrusion system in the CNS and appears to be involved in the development of NDDs such as ASD^30,31^. Protein analysis was performed on the whole lysate (total fraction) of the lumbar spinal cord at P8, followed by immunoblotting with specific antibodies against the KCC2 protein. The monomeric (i.e., inactive) and oligomeric (i.e., active) forms of KCC2 were detected at 140 kDa and > 250 kDa, respectively (Fig. 3B) (Supplementary Figure 1). For the inactive form, nonparametric, Kruskal–Wallis test indicated an effect of group (χ^2^ = 7.86; df = 3; p = 0.049). Post-hoc test, however, showed that the expression of the inactive form of KCC2 did not differ between groups (Fig. 3C). For the oligomeric or active form, Kruskal–Wallis test indicated no effect of group (χ^2^ = 5.06; df = 3; p = 0.17) (Fig. 3D). Thus, we found no beneficial impact of UC-MSC treatment on the expression of the active and inactive forms of KCC2, despite reduction of the hyperexcitability in the spinal cord. When the data in rats of the sham-vehicle and sham-MSC group were pooled together to form a larger group, the expression of the active oligomeric form was significantly lower in the LBW-vehicle group (W = 9; *p* = 0.049), compared to the pooled sham group, (Fig. 3D), but not in the LBW-MSC group (*p* = 0.37).

### Negative geotaxis

**Figure 4:**
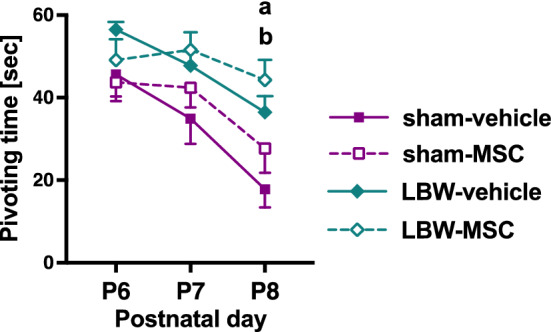
Negative geotaxis. Acquisition of the physiological reflex was evaluated using the negative geotaxis test from postnatal day 6 (P6) to P8. Pups turned upward faster as they aged. Data on male and female pups were combined. sham-vehicle (*n* = 12), sham-MSC (*n* = 15), LBW-vehicle (*n* = 24), and LBW-MSC (*n* = 13).a: *p* < 0.05, mice in the sham-vehicle vs. LBW-vehicle groups. b: *p* < 0.01, mice in the sham-vehicle vs. LBW-MSC groups. Mean ± standard error of mean. LBW, low birth weight; MSC, mesenchymal stromal cell; P6, postnatal day 6.

We performed a negative geotaxis test from P6 to P8 to evaluate the acquisition of sensorimotor reflex or the levels of neurodevelopment. Both male and female pups turned upward faster as they aged (Fig. 4). A significant difference was observed among the four groups (F_3, 60_ = 5.39, *p* = 0.002). Post-hoc tests revealed that pivoting times in pups of the LBW-vehicle and LBW-MSC groups on P8 were significantly longer than those in pups of the sham-vehicle group and that pivoting times did not significantly differ between pups of the LBW-vehicle and LBW-MSC groups. The pivoting times did not significantly differ between pups of the sham-vehicle and sham-MSC groups.

### Open-field activities

To evaluate the performance of spontaneous activities, we performed an open-field test at 3 and 6 weeks of age (equivalent to human early childhood and adolescence, respectively). We considered the total distance traveled as a hyperactivity indicator, and the duration of stay in the center zone was considered a lack of anxiety/inhibition indicator.

**Figure 5:**
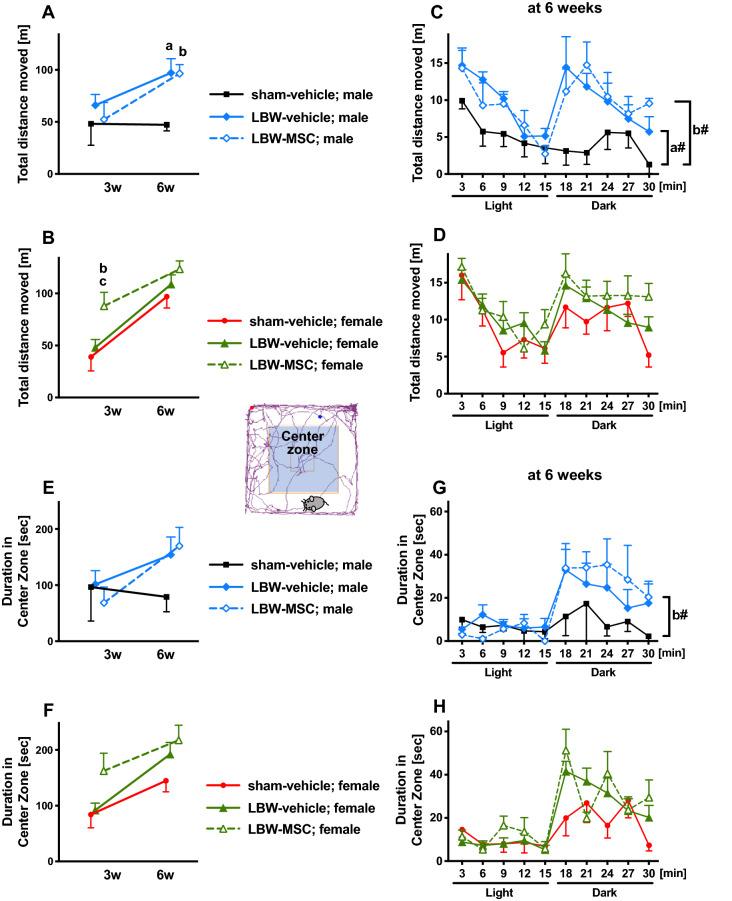
Open-field activities. Performance of spontaneous activities (total distance traveled) and anxiety/inhibition (duration of stay in the center zone) were evaluated using the open-field test during 30-min sessions at 3 and 6 weeks of age. A light environment was provided for the first 15 min, and a dark environment was provided for the subsequent 15 min. Total distance traveled during the 30-min session in males (**A**) and females (**B**). Temporal changes in the total distance traveled during the 30-min session in 3-min increments at 6 weeks of age in males (**C**) and females (**D**). Total duration of stay in the center zone during the 30-min session in males (**E**) and females (**F**). Temporal changes in the total duration of stay in the center zone during the 30-min session in 3-min increments at 6 weeks of age in males (**G**) and females (**H**). In the male group: sham-vehicle (*n* = 5), LBW-vehicle (*n* = 7), and LBW-MSC (*n* = 6). In the female group: sham-vehicle (*n* = 7), LBW-vehicle (*n* = 17), and LBW-MSC (*n* = 7). a: *p* < 0.05, mice in the sham-vehicle vs. LBW-vehicle groups. b: *p* < 0.05, mice in the sham-vehicle vs. LBW-MSC groups. c: *p* < 0.05, mice in the LBW-vehicle vs. LBW-MSC groups. # indicates the analyzed results for the 15-min dark environment. Mean ± standard error of mean. LBW, low birth weight; MSC, mesenchymal stromal cell.

Concerning the total distance traveled, we first analyzed the overall activities performed during 30-min sessions at 3 and 6 weeks. In males, no significant difference was observed among the sham-vehicle, LBW-vehicle, and LBW-MSC groups (F_2, 15_ = 2.18, *p* = 0.147) (Fig. 5A). However, post-hoc tests revealed that male pups in the LBW-vehicle and LBW-MSC groups, at 6 weeks of age, were significantly hyperactive compared to male pups in the sham-vehicle group. In females, there was a significant difference among the sham-vehicle, LBW-vehicle, and LBW-MSC groups (F_2, 28_ = 3.58, *p* = 0.041) (Fig. 5B). Although female pups in the LBW-MSC group were significantly hyperactive at 3 weeks of age compared to female pups in the sham-vehicle and LBW-vehicle groups, those in the LBW-MSC group were no longer hyperactive at 6 weeks of age compared to female pups in the sham-vehicle and LBW-vehicle groups. Second, at 6 weeks of age, we analyzed the total activities performed during 15 min in the light environment and 15 min in the dark environment. There was a significant group difference in males (F_2, 15_ = 6.21, *p* = 0.011) (Fig. 5C). Post-hoc tests revealed that males in the LBW-vehicle and LBW-MSC groups were significantly hyperactive compared to males in the sham-vehicle group in the dark, but not in the light environment. In females, there was no significant group difference (F_2, 28_ = 1.20, *p* = 0.317) (Fig. 5D).

Regarding the duration of stay in the center zone, we first analyzed the overall duration of stay during the 30-min sessions at 3 and 6 weeks. There were no significant group differences noted in the males (F_2, 15_ = 0. 468, *p* = 0.635) (Fig. 5E) or females (F_2, 28_ = 2.86, *p* = 0.074) (Fig. 5F). Second, at 6 weeks of age, we analyzed the duration of stay in the 15-min light environment and 15-min dark environment. In males, there were no significant group differences (F_2, 15_ = 2.12, *p* = 0.154) (Fig. 5G). However, in the dark environment, post-hoc tests revealed that males in the LBW-MSC group, but not those in the LBW-vehicle group (*p* = 0.1), stayed in the center zone significantly longer than males in the sham-vehicle group. In females, there were no significant group differences (F_2, 28_ = 1.62, *p* = 0.217) (Fig. 5H).

Taken together, LBW males, but not LBW females, exhibited hypermobility. Additionally, LBW males, but not LBW females, tended to exhibit less anxiety and disinhibition. MSC treatment had no significant impact in open-field testing.

### Three-chamber sociability

**Figure 6:**
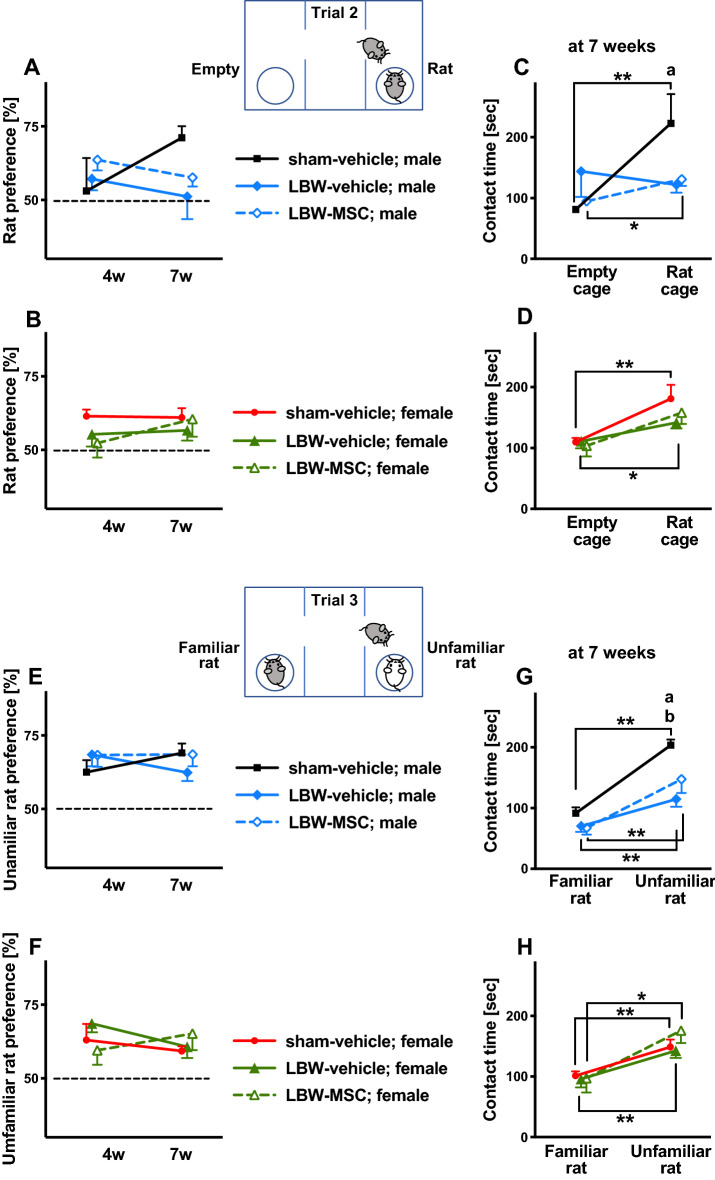
Three-chamber sociability. Sociability was evaluated using the three-chamber sociability test at 4 and 7 weeks of age. On the first day (trial 1), an examinee rat was acclimated in the three-chamber apparatus. On the second day (trial 2), an examinee rat was allowed to freely visit an empty cage and a cage with a rat. “Rat preference” was calculated as follows: [(time in contact with a rat cage)/(time in contact with a rat cage + an empty cage)] × 100%. Rat preference at 4 and 7 weeks of age in males (**A**) and females (**B**). Actual time in contact with an empty cage or a rat cage at 7 weeks of age in males (**C**) and females (**D**). On the third day (trial 3), an examinee rat was allowed to freely visit a familiar rat, i.e., a littermate bred in the same animal cage, and an unfamiliar rat. “Unfamiliar rat preference” was calculated as follows: [(time in contact with an unfamiliar rat)/(time in contact with an unfamiliar rat + a familiar rat)] × 100%. Unfamiliar rat preference at 4 and 7 weeks of age in males (**E**) and females (**F**). Actual time in contact with a familiar rat or an unfamiliar rat at 7 weeks of age in males (**G**) and females (**H**). In the male group: sham-vehicle (*n* = 5), LBW-vehicle (*n* = 7), and LBW-MSC (*n* = 6). In the female group: sham-vehicle (*n* = 7), LBW-vehicle (*n* = 17), and LBW-MSC (*n* = 7). Mean ± standard error of mean. a: *p* < 0.05, mice in the sham-vehicle vs. LBW-vehicle groups. b: *p* < 0.05, mice in the sham-vehicle vs. LBW-MSC groups. d: *p* < 0.05, mice in the LBW-vehicle vs. LBW-MSC groups. *p < 0.05, **p < 0.01 either empty vs. rat cage or familiar vs. unfamiliar rat. LBW, low birth weight; MSC, mesenchymal stromal cell.

We performed a three-chamber sociability test at 4 and 7 weeks of age. The rats were habituated to the apparatus on the first day (trial 1). Regarding the second day (trial 2), we analyzed the ratio of time spent in contact with the cage with a rat (rat cage) with time spent in contact with an empty cage, namely, the rat cage preference, at 4 and 7 weeks. No significant group difference was observed, i.e., among the sham-vehicle, LBW-vehicle, and LBW-MSC groups, in rat cage preference in males (F_2, 15_ = 1.03, *p* = 0.380) (Fig. 6A) or females (F_2, 28_ = 0.839, *p* = 0.443) (Fig. 6B). Subsequently, at 7 weeks of age, we analyzed the time spent in contact with the rat cage and the time spent in contact with an empty cage. Male rats in the sham-vehicle and LBW-MSC groups, but not those in the LBW-vehicle group, spent significantly more time in contact with the rat cage than in contact with an empty cage. Although no significant overall group difference was observed (F_2, 15_ = 1.47, *p* = 0.262), post-hoc tests revealed that rats in the LBW-vehicle group, but not in the LBW-MSC group, spent significantly less time in contact with the rat cage than rats in the sham-vehicle group (Fig. 6C). Female rats in the sham-vehicle and LBW-vehicle groups, but not in the LBW-MSC group (*p* = 0.067), spent significantly more time in contact with the rat cage than in contact with an empty cage. No significant group differences were observed (F_2, 28_ = 1.65, *p* = 0.210) (Fig. 6D).

Concerning the third day (trial 3), we analyzed the ratio of time spent in contact with an unfamiliar rat with the time spent in contact with a familiar rat, namely, unfamiliar rat preference, at 4 and 7 weeks. There was no significant group difference in unfamiliar rat preference in males (F_2, 15_ = 0.738, *p* = 0.495) (Fig. 6E) or females (F_2, 28_ = 0.355, *p* = 0.704) (Fig. 6F). At 7 weeks of age, we analyzed the time spent in contact with a familiar rat and an unfamiliar rat. In males, the three groups of rats spent significantly more time in contact with an unfamiliar rat than with a familiar rat. There was a significant overall group difference (F_2, 15_ = 6.56, *p* = 0.009) in male rats (Fig. 6G). Post-hoc tests revealed that males in the LBW-vehicle and LBW-MSC groups spent less time in contact with an unfamiliar rat than males in the sham-vehicle group. In females, the three groups spent significantly more time in contact with an unfamiliar rat than with a familiar rat. There was no significant group difference (F_2, 28_ = 0.615, *p* = 0.548) (Fig. 6H) in females.

Thus, LBW males, but not LBW females, exhibited disturbed sociability. MSC treatment ameliorated the disturbed sociability observed in trial 2, but not in trial 3.

### Cytokine levels in the serum and CSF

**Figure 7:**
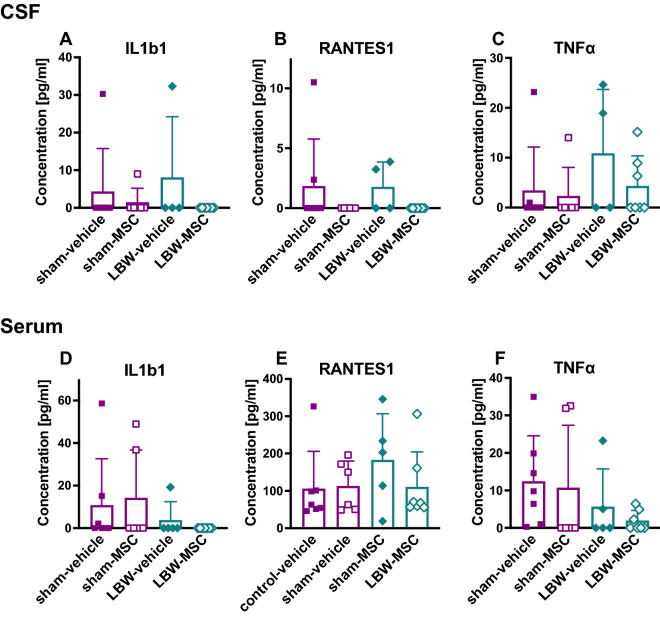
Cytokine levels. Cytokine levels were measured in the CSF (**A**–**C**) or serum (**D**–**F**), 24 h after vehicle or MSC injection. With respect to any cytokine measured, including IL-1b1, RANTES-1, and TNF-α1, there was no significant difference among the four groups, namely, sham-vehicle (*n* = 7), sham-MSC (*n* = 6), LBW-vehicle (CSF *n* = 4, serum *n* = 5), and LBW-MSC (*n* = 7). Data pertaining to males and females were combined. Mean ± standard deviation. CSF, cerebrospinal fluid; IL, interleukin; LBW, low birth weight; MSC, mesenchymal stromal cell; RANTES, regulated on activation normal T-cell expressed and secreted; TNF, tumor necrosis factor.

Twenty-four hours after MSC or vehicle injection in male and female pups, to evaluate the effects of MSC treatment on inflammation, we measured the expression levels of four cytokines: interleukin (IL) 1b1, Regulated on Activation, Normal T-cell Expressed and Secreted (RANTES)-1, tumor necrosis factor (TNF) α1, and interferon (IFN)-γ1. We found no significant difference in the CSF levels of any of these cytokines among the four groups of rats: IL1b1 (*p* = 0.625), RANTES-1 (*p* = 0.103), and TNFα1 (*p* = 0.547), using the Kruskal–Wallis test (Fig. 7A–C). Similarly, we found no significant difference in the serum levels of cytokines among the four groups: IL1b1 (*p* = 0.304), RANTES-1 (*p* = 0.557), and TNFα1 (*p* = 0.136) (Fig. 7D–F). Levels of IFN-γ1 were below detectable levels in the CSF and serum of most pups in the four groups.

### Neuronal counts in the whole hippocampus

**Figure 8:**
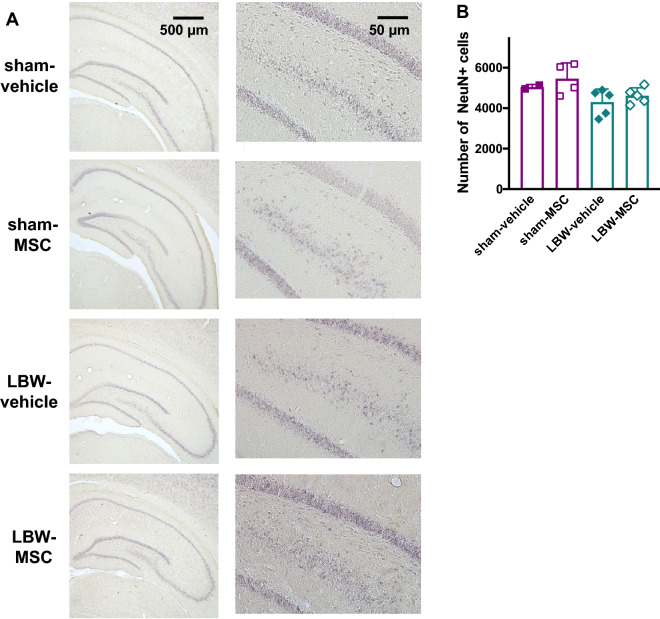
Neurons in the hippocampus. Representative images of the hippocampus in coronal sections stained with NeuN at 8 weeks of age (**A**). Shown on the right are high magnification images of the dentate gyrus. NeuN-positive cells were counted in the entire hippocampus in three coronal sections, and the averaged data are presented (**B**). There were no significant differences among groups. sham-vehicle (*n* = 2), sham-MSC (*n* = 4), LBW-vehicle (*n* = 5), and LBW-MSC (*n* = 5). Data pertaining to males and females were combined. Mean ± standard deviation. LBW, low birth weight; MSC, mesenchymal stromal cell.

LBW or MSC treatment had no impact on the relative brain weight of all groups. Thereafter, we investigated their effects on the neuronal counts in the whole hippocampus, which is involved in memory and cognitive functions. The neuronal counts among the four groups did not differ overall in the Kruskal–Wallis test (*p* = 0.13) (Fig. 8A,B). Of note, compared to rats in the pooled sham group, i.e., the sham-vehicle and sham-MSC groups combined, neuronal counts in the hippocampus were significantly lower in rats of the LBW-vehicle group, but not in rats of the LBW-MSC group.

## Discussion

To our knowledge, this is the first study to assess the impact of early administration of human UC-MSCs on physiology and behaviors associated with NDDs in a rat model of LBW based on intrauterine hypoperfusion. Our results indicate a positive effect of UC-MSC administration in reducing hyperexcitability in the lumbar spinal cord and a marginal positive effect on social interactions in males in the three-chamber test. Thus, UC-MSC treatment may improve different aspects of neurological problems in LBW children. We observed no significant effects of UC-MSC treatment in LBW rats on the (1) physical development of pups (body weight gain), (2) delay in acquiring a physiological reflex (negative geotaxis), (3) spontaneous hyperactivity and less anxiety-like behavior in the open-field test, and (4) reduced neuronal counts in the hippocampus, as observed in the LBW-vehicle rats. Preclinical studies performed in several types of animal models of postnatal brain injury, including hypoxia–ischemia^32,33^, stroke^34^, inflammation^35^, inflammation combined with hypoxia–ischemia^36,37^, and intraventricular hemorrhage^38^ have shown that human UC-MSCs have neuroprotective properties. However, few studies have been conducted in animal models of prenatal brain injuries^27,39^. With respect to ASD, some preclinical studies performed in genetically modified animal models for ASD^40^ or a valproate-induced ASD model^41^ have shown the beneficial effects of MSC treatment in autism-like behaviors. Therefore, the present study is the first to report some beneficial effects, although marginal, of MSCs derived from any source in an animal model of NDDs associated with LBW.

This study demonstrated that the MIUH model adequately simulated several neurological issues of LBW children. Our previous studies have shown that this model induced hyperactivity, delayed acquisition of a physiological reflex (negative geotaxis)^12^, and hyperexcitability in the spinal cord network^16^. For the first time, the present study demonstrated that this model also simulates disturbed sociability.

In developed countries, intrauterine hypoperfusion or ischemia and inflammation are considered as the major causes of LBW. Intrauterine hypoperfusion or ischemia induces sterile inflammation; thus, intrauterine inflammation may be the most important and convergent pathophysiology that leads to LBW^6,42,43^. Maternal immune activation during pregnancy plays a major role in the development of NDDs in human offspring^44^, regardless of whether they have LBW. Our previous studies revealed that this MIUH rat model exhibited intrauterine inflammation^12^. However, the degree of inflammation is mild; levels of inflammatory cytokines are elevated only in the placenta and not in the fetal brain, amniotic fluid, or dam’s serum^12^, and the percentage of Iba1-positive cells (microglia) did not increase when evaluated in adulthood^45^. The present study showed that the levels of inflammatory cytokines in the CSF and serum during the neonatal period were not elevated in pups with MIUH-induced LBW. We expected MSC treatment to decrease pro-inflammatory cytokine levels, but the treatment effects in the levels of these inflammatory parameters were not significant, which could partly be due to the fact that the model exhibited no marked increases in cytokine levels, and primarily due to the small sample size. Other studies using comparable MIUH models showed various levels of inflammation and oxidative stress in young and adult offspring^13–15^. A more detailed analysis on inflammation is needed to examine the hypothesized immunomodulatory effect of MSC treatment.

To our knowledge, the present study is the first to show that early treatment with UC-MSC reduced hyperexcitability in the lumbar spinal cord of LBW animals, which is a sign of spasticity^16^, evidenced by the restoration of post-activation depression corresponding to the level of sham animals. At least two mechanisms have been proposed for such hyperreflexia: increased excitability of motor neurons and reduced inhibition (the so-called disinhibition) within the spinal network^46^. Disinhibition of the stretch reflex seems to be related to the reduced expression of the active form of KCC2, which atypically increases the intracellular concentration of chloride and reverses the effect of gamma-aminobutyric acid from hyperpolarization to depolarization^46,47^, thereby leading to hyperreflexia and signs of spasticity, as we had reported previously^16^. Postmortem cerebral samples of human preterm infants with white matter injury showed reduced expression of KCC2, suggesting the involvement of KCC2 and cell excitability in the emergence of NDDs^48^. The early administration of UC-MSC induced a non-significant (likely related to small sample size) near-restoration of the expression of the active oligomeric form of KCC2, which may explain the attenuation of spinal hyperexcitability found in rats of the LBW-vehicle group. MSC application in cultured hippocampal cells increased the expression of KCC2 and inhibitory transmission via brain-derived neurotrophic factor production^49^. Recently, Cao et al.^50^ showed that transplantation of UC-MSCs after spinal cord injury upregulated KCC2 expression and promoted the recovery of motor-evoked potentials. Other studies have reported the deleterious contribution of excess calpain activity to the cleavage and inactivation of KCC2, thereby contributing to hyperexcitability. Calpains are intracellular proteases that are activated by calcium influx during excitotoxicity and inflammatory processes^19,20^. We propose that early administration of UC-MSCs may regulate calpain activity, tending to the restoration of KCC2 expression and reduced hyperexcitability in offspring after MIUH.

Apart from their suppressive effects on inflammation and spinal hyperexcitability, UC-MSCs possess multifaceted effects on prenatal and neonatal brain injuries, as follows: (1) secreting trophic factors and extracellular vesicles^37,38^; (2) ameliorating altered levels of brain metabolites^51^; (3) improving brain energy metabolism^33^; (4) attenuating hypomyelination and decreasing the number of oligodendrocytes^33,38^; (5) attenuating reactive gliosis^38,39^; and (6) attenuating apoptosis and cell death^33,38^.

Although we hypothesized that UC-MSC treatment would ameliorate hypermobility in LBW rats, this was not observed. Similar to the results of the present study, in previous studies, bone marrow-derived (BM)-MSC treatment did not alter the total distance traveled and duration of stay in the center zone in an open-field test, although the treatment ameliorated social behaviors in genetically modified and valproate-induced mouse models of ASD^41,52^. Delayed acquisition of physiological neonatal reflex observed in the negative geotaxis test in the LBW pups was not ameliorated by UC-MSC treatment in the present study. These results do not necessarily indicate a lack of beneficial effects of UC-MSCs on hypermobility and acquisition of the reflex. We tested only one treatment protocol, a single dose, intravenous injection of 10^5^ cells at P1, in this study. This protocol was based on our previous studies that had demonstrated beneficial effects on behavior, inflammation, and metabolism in the brain in a mouse model of neonatal stroke, in which a single dose of 10^4^ cells or 10^5^ cells were intravenously injected at P14^27,34,51^. Other treatment protocols may exert beneficial effects in this intrauterine hypoperfusion model. As our previous study demonstrated dose-dependent effects^34^, higher doses may exert beneficial effects in this model as well. Repeated UC-MSC administration may also enhance the beneficial effect as recent studies show that repeated cell administrations are more beneficial than a single cell administration^53,54^.

This study had the following limitations. First, the mechanism of action of the amelioration of disturbed sociability was not explored. On one hand, subtle brain damage with no obvious neuronal cell loss or neuroinflammation in this model may be hinder the evaluation of treatment effects and the mechanism of action. On the other hand, such subtle damage may establish an optimal model to evaluate the pathophysiology of NDDs and treatment effects because it accurately simulates the actual condition of LBW children with NDDs. Not all LBW children develop NDDs, and not all LBW children with NDDs have maternal intrauterine inflammation as the primary cause of LBW and NDDs^2^. Majority of children with NDDs exhibit subtle, but not obvious, abnormalities in imaging studies or markers of inflammation^44,55,56^. Our model was designed to simulate such real-life conditions, therefore, it demonstrated modest histological brain damage (i.e., non-significant reduction of neuronal counts), mild behavioral phenotype, and weak inflammation (i.e., non-significant changes in cytokine levels in the serum or CSF). Other models with intrauterine ischemia exhibit more pronounced morphological brain damage and systemic and cerebral inflammation^27,57^. The very mild model of LBW with NDDs used in this study may be an optimal model for evaluating the realistic effects of novel treatments before clinical translation, but may not be optimal for exploring the mechanisms of action of the treatments, especially in studies with relatively small sample size. We previously reported that a comparable but more progressive intrauterine hypoperfusion model showed neuroinflammation (increased Iba1-positive cells) and reduced neuronal counts at 8 weeks of age^27^. In this model, intravenous administration of human UC-MSCs ameliorated the decrease in neuronal counts and increased the number of M2 microglia, which resulted in an anti-inflammatory effect^27^. Studies in genetically modified and valproate-induced mouse models of ASD showed that intracerebral BM-MSC transplantation increases hippocampal neurogenesis^41,52^.

In summary, intravenous administration of human UC-MSCs in the early postnatal period ameliorated spinal hyperexcitability and tended to improve sociability, but not the other examined parameters. The overall beneficial effects of UC-MSC treatment were unremarkable; hence, the treatment protocol, for example, cell dose, timing, and treatment frequency, may need to be optimized. Considering the limited preventive measures for NDDs and cerebral palsy in LBW children, we believe that the beneficial effects of UC-MSCs are worth exploring for possible clinical translation.

## Materials and methods

All the methods used on animals were performed in accordance with the relevant guidelines and regulations: the National Institutes of Health publication number 80-23, the European Council Directive (2010/63/EEC), and the Animal Research: Reporting of In Vivo Experiments (ARRIVE) guidelines (https://arriveguidelines.org). This research involving animals was approved by the local and national committees in France (Autorisation de Projet utilisant des Animaux à des Fins Scientifiques [APAFIS] authorization number 30976-2021040915234664) and Japan (Kyoto Women’s University Animal Research Ethics Committee certificate number 30-3). This research involving human materials, i.e., human UC-MSCs, was approved by the Ethics Committee of the Institute of Medical Science, the University of Tokyo (IMSUT), and Kyoto Women’s University, Institut de Neurosciences de la Timone, Centre National de la Recherche Scientifique, France. UC-MSCs were provided by cord blood and umbilical cord bank from the research hospital, IMSUT (IMSUT CORD), Japan (Ethical committee of IMSUT number 33-2).

### Intrauterine arterial stenosis using microcoils

In previous studies, intrauterine artery stenosis was performed using unilateral ligation on E17 to induce prenatal ischemia and FGR in rats^9–11,58^. We developed a novel model of intrauterine ischemia/growth retardation by wrapping metal-coated coils (Samini Co. Ltd., Shizuoka, Japan) around the bilateral intrauterine arteries of the dams on E17^12,59^. Briefly, under deep anesthesia with isoflurane, microcoils (inner diameter: 0.24 mm) were wrapped around the proximal parts of the ovarian and vaginal arteries using a previously described procedure^12,59^, to induce adequate blood flow reduction or MIUH (Fig. 1). This technique has the advantage of resulting to more numbers of LBW pups, thereby reducing the number of dams required compared to that required in stenosis by ligation. The sham group for electrophysiology and Western blot tests was subjected to the same surgery as the MIUH group but without coil application, while the sham group for other experiments was subjected to anesthesia without laparotomy on E17^12^. Pups were delivered by spontaneous labor, and depending on birth weight, were assigned to a specific group. According to the criteria used in previous studies, pups weighing < 5.5 g were considered to exhibit growth retardation and were included in the LBW group^10,16,58^.

### Laser speckle blood flowmetry

We measured the temporal changes in intrauterine blood flow under isoflurane anesthesia (2.0% isoflurane) using laser speckle flowmetry (Omegazone, Omegawave Inc., Tokyo, Japan) at three time points: before stenosis, 1 h, and 3 days after stenosis on both ovarian and vaginal sides (Fig. 1). To quantify blood flow in all fetuses or placentas, regions of interest were determined in two sham and two MIUH rats (Fig. 1A,B).

### UC-MSC administration

We obtained UCs from pregnant women immediately after delivery. Written informed consent was obtained from all patients before delivery. The UCs were treated and cultured for four passages as previously described^38^. The collected UC-MSCs were frozen at − 80 °C with a cryoprotectant (STEM-CELL BANKER, ZENOAQ Resource Co. Ltd.). On P1, pups were anesthetized using isoflurane inhalation, and the skin over the facial vein was cut. UC-MSCs were thawed, and the concentration of the cell solution was adjusted by adding phosphate-buffered saline (PBS). The cell solution was injected under a microscope at a concentration of 10^5^ cells/60 μL in the facial vein in the sham-MSC and LBW-MSC groups. Similarly, pups in the sham-vehicle and LBW-vehicle groups received 60 μL of a vehicle solution.

### Early in vitro post-activation depression

We assessed alterations in the monosynaptic reflex loop using an in vitro whole spinal cord preparation on P5 to P6 to determine the early impact of MIUH on functional reorganization and excitation/inhibition balance in rat pups. The spinal cord below T8 was isolated from neonatal rats between P4 and P6, as previously described^16,46^, and transferred to a recording chamber perfused with oxygenated (95% O_2_/5% CO_2_) CSF composed of the following (in mM): NaCl 130, KCl 4, CaCl_2_ 3.75, MgSO_4_ 1.3, NaH_2_PO_4_ 0.58, NaHCO_3_ 25, and glucose 10 (pH 7.4; 24–26 °C). Extracellular stimulation and recording were performed at the level of ventral (VR5) and dorsal (DR5) roots of L5 by touching them with stainless steel electrodes insulated with petroleum jelly. AC recordings from the VR5 were amplified (× 2000) and bandpass-filtered between 70 Hz and 3 kHz. Supramaximal stimulation of the DR5 elicited a monosynaptic response in the ipsilateral homonymous VR5 in vitro*,* corresponding to the earliest component of motor neuron excitation. To determine the level of post-activation depression at different frequencies, we discarded responses to the first three stimulations required for the occurrence of depression. The responses were rectified, and the areas under the curves were measured. The monosynaptic response was expressed in percentage relative to the mean response at 0.1 Hz in the same series of measurements^16^.

### Western blot analysis for postnatal expression of KCC2, a cotransporter of chloride

To detect the expression of KCC2 in the spinal cord, tissues were collected at P8 and frozen after removing the dorsal and ventral roots. Samples were prepared in an ice-cold lysis buffer containing 1% Igepal CA-630 (Sigma-Aldrich, Merck KGaA, Germany), 0.1% sodium dodecyl sulfate, 10 mM sodium vanadate, 10 mM sodium fluoride, 10 mM sodium pyrophosphate, and 1.8 mg/mL iodoacetamide supplemented with a cocktail of protease inhibitors (Complete-mini, Roche Life Science). After centrifugation at 18,000×*g* for 30 min at 4 °C, the supernatant was collected, and protein concentrations were determined using the DC protein assay (BioRad). Proteins of equivalent amounts (30 µg) were separated from the samples by performing electrophoresis using SDS-PAGE (4–15% Criterion™ TGX Stain-Free™ Precast Gels, BioRad). These were transferred to a nitrocellulose membrane and incubated overnight at 4 °C with the affinity-purified rabbit anti-KCC2 polyclonal antibody (diluted 1:1000; Merck-Millipore). The blot was then incubated after 1 h at 24 °C with an ImmunoPure goat horseradish peroxidase-conjugated rabbit-specific antibody (1:80,000, Thermo Scientific, in blocking solution of Tris-buffered saline containing 5% fat-free milk powder). Bands were visualized using chemiluminescence (Merck-Millipore). Signal intensities were measured using ImageQuant LAS 4000 and ImageQuant LAS 4000 Control Software (GE HealthCare). Equal amounts of protein samples were loaded, and total protein normalization was performed using stain-free imaging (BioRad), which makes the proteins fluorescent directly in the gel, followed by their transfer. The total density for each lane was measured from the blot and used to calculate the normalizing factors. After normalization to the total protein signal intensity for KCC2, we normalized the data by dividing each sample by the mean value of the control samples^60^.

### Behavioral assessments of early NDDs

#### Negative geotaxis

A negative geotaxis test was performed for three consecutive days from P6 to P8, as previously reported, to evaluate the development of the sensorimotor reflex^12^. The days on which the test was performed were adjusted such that the post-surgery days were the same for each pup. For example, adjusted P6 was P6 for pups born on E22 and P7 for pups born on E21. Each pup was placed on a board inclined at an angle of 25° with their heads facing downward. The time required for each pup to turn upward on the board (i.e., 180°) was recorded. The trial was terminated after 60 s.

#### Open-field testing

An open-field test was performed at 3 and 6 weeks of age to evaluate spontaneous activity. The rats were allowed to freely move in a box (100 × 100 × 40 cm) for 15 min in a light environment and for the subsequent 15 min in a dark environment. The rat movements were traced using AnyMaze, an automated video tracking system (Stoelting Co., Wood Dale, IL, USA). The test was performed between 10 a.m. and 5 p.m.

#### Three-chamber social test

To evaluate sociability (interaction with other rats), the three-chamber social test was performed at 4 and 7 weeks of age. A rat was placed in a box (60 × 40 × 30 cm) separated into three compartments or chambers, which the rat could freely visit and leave, for 10 min for three consecutive days. On the first day (trial 1), the rat was acclimated in the three-chamber box. On the second day (trial 2), a same-sex stimulus rat, which was familiar to the examinee rat, was placed in a wired cage in the right chamber; an empty cage was placed in the left chamber, and the examinee rat was placed in the middle chamber. On the third day (trial 3), a same-sex “unfamiliar” rat was placed in the wired cage in the right chamber, and a same-sex “familiar” rat, which was a littermate of the examinee rat raised in the same breeding cage, was placed in a wired cage in the left chamber; the examinee rat was placed in the middle chamber. The movement of the examinee rat was traced using AnyMaze, and the time in contact with other rats in the wired cages was measured.

Although all four groups were tested for the open-field test and three-chamber sociability test, the number of males and female pups in the sham-MSC group was insufficient and a statistical analysis of the behavioral test could not be performed. Therefore, only the data from the remaining three groups were analyzed.

### Cytokine expression

The levels of several cytokines were measured in serum and CSF to evaluate the early effects of MSC treatment on inflammation. Twenty-four hours after the injection (P2), CSF was collected by puncturing the cisterna magna with a 25-gauge needle and a capillary glass under a microscope. An early time point for cytokine level measurement was chosen because (1) our previous study showed that increased levels of many cytokines decrease within 24 h after hypoxic-ischemic insult in pups^61^ and (2) our other study showed that injected MSCs do not survive for a long duration, suggesting that the beneficial effects of MSC treatment might be due to suppression of inflammation in the early phase^34^. Blood was collected by cardiac puncture and centrifuged to obtain the serum. Males and females were analyzed together. The concentrations of the following cytokines were measured using a bead immunoassay as previously described^38^: IFN-γ1, IL-1b1, RANTES-1, and TNF α1. All samples were analyzed in triplicate, according to the manufacturer’s instructions. Bead fluorescence readings were recorded using a BD™ FACS Canto II flow cytometer (BD Biosciences, San Jose, CA).

### Neuronal counts

Neuronal counts were performed in the entire hippocampi of the sham and LBW offspring at 8 weeks of age, as previously described^27^. After deep anesthesia, the rats were intracardially perfused with PBS solution and then with 4% paraformaldehyde in PBS. Whole brains were collected and immersed in the same paraformaldehyde solution overnight. These were successively transferred to buffer solutions containing 10%, 20%, and 30% sucrose; frozen with powdered dry ice; and stored at − 80 °C. Forty-micrometer coronal sections were subsequently cut at three hippocampal levels, which were determined using a rat brain atlas (at − 2.76 mm, − 4.20 mm, and − 5.64 mm caudal to the bregma^55^). The sections were blocked with 0.6% H_2_O_2_ in PBS and then with 3% normal donkey serum and 0.1% Triton-X100 (Sigma-Aldrich, Merck KGaA, Germany) in PBS for 30 min. The sections were incubated overnight at 4 °C with a primary antibody (mouse anti-NeuN, 1:400, clone A60, EMD Millipore, USA) in 3% donkey serum and PBS. The sections were further incubated on the second day with a secondary antibody (1:1000; biotinylated donkey anti-mouse; Jackson ImmunoResearch Laboratories, USA) in 3% donkey serum and 0.1% Triton-X100 in PBS for 60 min. After using Avidin–Biotin-Peroxidase (Vectastain Elite ABC Kit; Vector Laboratories, USA) for 60 min, peroxidase detection was performed for 15 min (0.25 mg/mL DAB, 0.01% H_2_O_2_, and 0.04% NiCl_2_). NeuN-positive cells, i.e., the nuclei of neurons firmly stained with NeuN antibody, in the three coronal sections in the entire hippocampus (CA1-3 and dentate gyrus, including dorsal and ventral hippocampus) were counted using unbiased counting techniques^56^ (Stereo Investigator version 10 stereology software, Micro Bright Field Europe EK, Magdeburg, Germany). After outlining the borders of the hippocampus, the computer program overlaid the outlined area with a grid system for counting the frames. NeuN-positive cells within these frames, as well as those touching two out of four predetermined sides of the frames, were counted by gradually shifting the focus along the Z axis. The average values of the three sections were used for analysis.

### Statistical analysis

Statistical analyses were performed using R^62^ or Prism 8 (GraphPad Software, USA). Birth weight, body weight at P14, brain/body weight ratio, and neuronal counts were analyzed using one-way ANOVA followed by Tukey’s test. Temporal changes in body weight and data from the electrophysiology, negative geotaxis, open-field, and three-chamber sociability tests (except data for contact time) within each group were analyzed using one-way repeated measures ANOVA followed by Tukey’s test. Contact time within each group from the three-chamber sociability test was analyzed using a paired *t*-test. Nonparametric Kruskal–Wallis test followed by Dunn’s test was used for Western blot analysis, cytokine level analysis, and neuronal counting. The results of the behavioral tests were expressed as means ± standard errors of the mean. Other results were expressed as means ± standard deviations. The significance level was set at *p* < 0.05. Although we analyzed the four groups together, only the statistically significant differences among the three groups, i.e., the sham-vehicle, LBW-vehicle, and LBW-MSC groups, and those between the two sham groups, i.e., the sham-vehicle and sham-MSC, are shown in figures.

## Supplementary Information

Supplementary Figure 1.

## Data Availability

The datasets generated and/or analyzed during the current study are available from the corresponding author upon reasonable request.

## References

[1] von Beckerath A-K (2013). Perinatal complications and long-term neurodevelopmental outcome of infants with intrauterine growth restriction. Am. J. Obstet. Gynecol..

[2] Sucksdorff M (2015). Preterm birth and poor fetal growth as risk factors of attention-deficit/hyperactivity disorder. Pediatrics.

[3] Unicef. Monitoring the situation of children and women: Low birthweight. *UNICEF DATA* https://data.unicef.org/topic/nutrition/low-birthweight/ (2019).

[4] Pascal A (2018). Neurodevelopmental outcome in very preterm and very-low-birthweight infants born over the past decade: A meta-analytic review. Dev. Med. Child Neurol..

[5] Robinson R (2020). Mental health outcomes of adults born very preterm or with very low birth weight: A systematic review. Semin. Fetal. Neonatal Med..

[6] Hagberg H (2015). The role of inflammation in perinatal brain injury. Nat. Rev. Neurol..

[7] Fleiss B, Gressens P, Stolp HB (2020). Cortical gray matter injury in encephalopathy of prematurity: Link to neurodevelopmental disorders. Front. Neurol..

[8] Ophelders DRMG (2020). Preterm brain injury, antenatal triggers, and therapeutics: Timing is key. Cells.

[9] Delcour M (2011). Mild musculoskeletal and locomotor alterations in adult rats with white matter injury following prenatal ischemia. Int. J. Dev. Neurosci..

[10] Delcour M (2012). Neuroanatomical, sensorimotor and cognitive deficits in adult rats with white matter injury following prenatal ischemia. Brain Pathol..

[11] Delcour M (2012). Impact of prenatal ischemia on behavior, cognitive abilities and neuroanatomy in adult rats with white matter damage. Behav. Brain Res..

[12] Ohshima M (2016). Mild intrauterine hypoperfusion reproduces neurodevelopmental disorders observed in prematurity. Sci. Rep..

[13] Yin S, Wang Y, Meng Y-L, Liu C-X (2020). Effects of mild intrauterine hypoperfusion in the second trimester on memory and learning function in rat offspring. Neural Regen. Res..

[14] Yin S, Meng Y, Liu C, Wang Y (2021). MIUH inhibits the hippocampal neuron growth in fetal rat by affecting the PTEN pathway. Neurochem. Res..

[15] Rains ME (2021). Oxidative stress and neurodevelopmental outcomes in rat offspring with intrauterine growth restriction induced by reduced uterine perfusion. Brain Sci..

[16] Coq JO (2018). Mild intrauterine hypoperfusion leads to lumbar and cortical hyperexcitability, spasticity, and muscle dysfunctions in rats: Implications for prematurity. Front. Neurol..

[17] Coq J-O (2020). From cerebral palsy to developmental coordination disorder: Development of preclinical rat models corresponding to recent epidemiological changes. Ann. Phys. Rehabil. Med..

[18] Blaesse P (2006). Oligomerization of KCC2 correlates with development of inhibitory neurotransmission. J. Neurosci. Off. J. Soc. Neurosci..

[19] Jantzie LL, Winer JL, Corbett CJ, Robinson S (2016). Erythropoietin modulates cerebral and serum degradation products from excess calpain activation following prenatal hypoxia-ischemia. Dev. Neurosci..

[20] Jantzie LL (2014). Erythropoietin attenuates loss of potassium chloride co-transporters following prenatal brain injury. Mol. Cell. Neurosci..

[21] Van Steenwinckel J (2014). Brain damage of the preterm infant: new insights into the role of inflammation. Biochem. Soc. Trans..

[22] Jung E (2020). The fetal inflammatory response syndrome: the origins of a concept, pathophysiology, diagnosis, and obstetrical implications. Semin. Fetal. Neonatal Med..

[23] Fleiss B (2021). Microglia-mediated neurodegeneration in perinatal brain injuries. Biomolecules.

[24] Juul SE (2020). A randomized trial of erythropoietin for neuroprotection in preterm infants. N. Engl. J. Med..

[25] Cunningham CJ, Redondo-Castro E, Allan SM (2018). The therapeutic potential of the mesenchymal stem cell secretome in ischaemic stroke. J. Cereb. Blood Flow Metab. Off. J. Int. Soc. Cereb. Blood Flow Metab..

[26] Jantzie LL, Scafidi J, Robinson S (2018). Stem cells and cell-based therapies for cerebral palsy: a call for rigor. Pediatr. Res..

[27] Kitase Y (2020). Establishment of a novel fetal growth restriction model and development of a stem-cell therapy using umbilical cord-derived mesenchymal stromal cells. Front. Cell. Neurosci..

[28] Nagamura-Inoue T, He H (2014). Umbilical cord-derived mesenchymal stem cells: Their advantages and potential clinical utility. World J. Stem Cells.

[29] Mukai T, Tojo A, Nagamura-Inoue T (2018). Mesenchymal stromal cells as a potential therapeutic for neurological disorders. Regen. Ther..

[30] Ben-Ari Y (2015). Commentary: GABA depolarizes immature neurons and inhibits network activity in the neonatal neocortex in vivo. Front. Cell. Neurosci..

[31] Anacker AMJ (2019). Enhanced social dominance and altered neuronal excitability in the prefrontal cortex of male KCC2b mutant mice. Autism Res. Off. J. Int. Soc. Autism Res..

[32] Zhu L-H (2014). Improvement of human umbilical cord mesenchymal stem cell transplantation on glial cell and behavioral function in a neonatal model of periventricular white matter damage. Brain Res..

[33] Robertson NJ (2021). Human umbilical cord mesenchymal stromal cells as an adjunct therapy with therapeutic hypothermia in a piglet model of perinatal asphyxia. Cytotherapy.

[34] Tanaka E (2018). Dose-dependent effect of intravenous administration of human umbilical cord-derived mesenchymal stem cells in neonatal stroke mice. Front. Neurol..

[35] Morioka C (2017). Neuroprotective effects of human umbilical cord-derived mesenchymal stem cells on periventricular leukomalacia-like brain injury in neonatal rats. Inflamm. Regen..

[36] Thomi G, Surbek D, Haesler V, Joerger-Messerli M, Schoeberlein A (2019). Exosomes derived from umbilical cord mesenchymal stem cells reduce microglia-mediated neuroinflammation in perinatal brain injury. Stem Cell Res. Ther..

[37] Thomi G (2019). Intranasally administered exosomes from umbilical cord stem cells have preventive neuroprotective effects and contribute to functional recovery after perinatal brain injury. Cells.

[38] Mukai T (2017). Intravenous injection of umbilical cord-derived mesenchymal stromal cells attenuates reactive gliosis and hypomyelination in a neonatal intraventricular hemorrhage model. Neuroscience.

[39] Paton MCB (2019). Umbilical cord blood versus mesenchymal stem cells for inflammation-induced preterm brain injury in fetal sheep. Pediatr. Res..

[40] Perets N (2017). Long term beneficial effect of neurotrophic factors-secreting mesenchymal stem cells transplantation in the BTBR mouse model of autism. Behav. Brain Res..

[41] Gobshtis N, Tfilin M, Wolfson M, Fraifeld VE, Turgeman G (2017). Transplantation of mesenchymal stem cells reverses behavioural deficits and impaired neurogenesis caused by prenatal exposure to valproic acid. Oncotarget.

[42] Driscoll DJO, Felice VD, Kenny LC, Boylan GB, O’Keeffe GW (2018). Mild prenatal hypoxia-ischemia leads to social deficits and central and peripheral inflammation in exposed offspring. Brain. Behav. Immun..

[43] Gall AR, Amoah S, Kitase Y, Jantzie LL (2022). Placental mediated mechanisms of perinatal brain injury: Evolving inflammation and exosomes. Exp. Neurol..

[44] Han VX, Patel S, Jones HF, Dale RC (2021). Maternal immune activation and neuroinflammation in human neurodevelopmental disorders. Nat. Rev. Neurol..

[45] Itoh A (2022). Bifidobacterium breve during infancy attenuates mobility in low birthweight rats. Pediatr. Int..

[46] Gackière F, Vinay L (2015). Contribution of the potassium-chloride cotransporter KCC2 to the strength of inhibition in the neonatal rodent spinal cord in vitro. J. Neurosci..

[47] Boulenguez P (2010). Down-regulation of the potassium-chloride cotransporter KCC2 contributes to spasticity after spinal cord injury. Nat. Med..

[48] Robinson S, Mikolaenko I, Thompson I, Cohen ML, Goyal M (2010). Loss of cation-chloride cotransporter expression in preterm infants with white matter lesions: Implications for the pathogenesis of epilepsy. J. Neuropathol. Exp. Neurol..

[49] Mauri M (2012). Mesenchymal stem cells enhance GABAergic transmission in co-cultured hippocampal neurons. Mol. Cell. Neurosci..

[50] Cao T (2022). hUC-MSC-mediated recovery of subacute spinal cord injury through enhancing the pivotal subunits β3 and γ2 of the GABAA receptor. Theranostics.

[51] Tanaka E (2020). Metabolomic analysis and mass spectrometry imaging after neonatal stroke and cell therapies in mouse brains. Sci. Rep..

[52] Segal-Gavish H (2016). Mesenchymal stem cell transplantation promotes neurogenesis and ameliorates autism related behaviors in BTBR mice. Autism Res. Off. J. Int. Soc. Autism Res..

[53] Penny TR (2020). Multiple doses of umbilical cord blood cells improve long-term brain injury in the neonatal rat. Brain Res..

[54] Magota H (2021). Repeated infusion of mesenchymal stem cells maintain the condition to inhibit deteriorated motor function, leading to an extended lifespan in the SOD1G93A rat model of amyotrophic lateral sclerosis. Mol. Brain.

[55] Lord C (2020). Autism spectrum disorder. Nat. Rev. Dis. Primer.

[56] AlbajaraSáenz A, Villemonteix T, Massat I (2019). Structural and functional neuroimaging in attention-deficit/hyperactivity disorder. Dev. Med. Child Neurol..

[57] Jantzie LL, Robinson S (2015). Preclinical models of encephalopathy of prematurity. Dev. Neurosci..

[58] Olivier P, Baud O, Evrard P, Gressens P, Verney C (2005). Prenatal ischemia and white matter damage in rats. J. Neuropathol. Exp. Neurol..

[59] Tsuji M, Coq JO, Ogawa Y, Yamamoto Y, Ohshima M (2018). A rat model of mild intrauterine hypoperfusion with microcoil stenosis. J. Vis. Exp..

[60] Caravagna C (2022). Prenatal hypoxia induces Cl^−^ cotransporters KCC2 and NKCC1 developmental abnormality and disturbs the influence of GABAA and glycine receptors on fictive breathing in a newborn rat. Front. Physiol..

[61] Ogawa Y, Tanaka E, Sato Y, Tsuji M (2021). Brain damage caused by neonatal hypoxia-ischemia and the effects of hypothermia in severe combined immunodeficient (SCID) mice. Exp. Neurol..

[62] R Core Team. *R: A Language and Environment for Statistical Computing*.

