# Supplementary material for: Umbilical cord-derived mesenchymal stromal cell therapy to prevent the development of neurodevelopmental disorders related to low birth weight
Source: Sci Rep. 2023 Mar 7;13:3841. doi: 10.1038/s41598-023-30817-3 (PMC9992354; doi:10.1038/s41598-023-30817-3)
Supplement: Supplementary file 1 — Supplementary Figure 1. [file 41598_2023_30817_MOESM1_ESM.docx]

**Supplemental Figure 1**

**Legend of Supplemental Figure 1**

Total protein level after gel loading and transfer to polyvinylidene fluoride membrane. The total protein amount was detected by Stain-Free technology after transfer. Stain-Free gels (Bio-Rad) contain a trihalocompound within the gel matrix that produces a fluorescent product when covalently crosslinked to protein tryptophan residues. To activate the stain-free gel, the gel was exposed to UV light for 1 minute using Chemidoc XRS (Biorad) imaging system equipped with a UV illumination source. Immediately after the transfer was completed, protein bands have been detected using the same imaging system. The right column corresponds to the molecular weight marker with ß-tubulin.

Band density analysis was performed for all images using image J free software. ß-tubulin is used as a reporter protein. The intensity of the bands was normalized by the intensity of the corresponding ß-tubulin band for each membrane in the present study. Images were transformed to 8-bit, then brightness and contrast were adjusted equally on the entire image, as depicted in the figure 3C.
